# The Diamond Disk

**Published:** 1877-06

**Authors:** 


### THE DIAMOND DISK.

We received recently from Dr. Babcock what he denominates an improved disk, for separating and dressing the teeth. The improvement consists in having the diamond dust fixed upon the edge of the disk as well as upon the sides, and the dust is brought more into and through the copper, than those made heretofore,—though the disks that were first made and those we have been using for six months, seem not the least impaired.

These disks certainly have no equal for durability and efficiency of operations. They should be in the hands of every good operator.

